# The Inhibitory Effect of Ellagic Acid on Cell Growth of Ovarian Carcinoma Cells

**DOI:** 10.1155/2013/306705

**Published:** 2013-06-16

**Authors:** Yuan-Chiang Chung, Li-Cheng Lu, Ming-Hsiu Tsai, Yu-Jen Chen, Yi-Ying Chen, Shih-Ping Yao, Chih-Ping Hsu

**Affiliations:** ^1^Department of Surgery, Cheng-Ching Hospital, Chung-Kang Branch, No. 118, Section 3, Chung Kang Road, Taichung City 40764, Taiwan; ^2^Department of Obstetrics and Gynecology, Taoyuan General Hospital, Department of Health, No. 1492, Chung-Shan Road, Taoyuan City 33004, Taiwan; ^3^Department of Medical Laboratory Science and Biotechnology, Yuanpei University, No. 306 Yuanpei Street, Hsinchu City 30015, Taiwan

## Abstract

Ellagic acid (EA) is able to inhibit the growth of several cancer cells; however, its effect on human ovarian carcinoma cells has not yet been investigated. Ovarian carcinoma ES-2 and PA-1 cells were treated with EA (10*~*100 **μ**M) and assessed for viability, cell cycle, apoptosis, anoikis, autophagy, and chemosensitivity to doxorubicin and their molecular mechanisms. EA inhibited cell proliferation in a dose- and time-dependent manner by arresting both cell lines at the G1 phase of the cell cycle, which were from elevating p53 and Cip1/p21 and decreasing cyclin D1 and E levels. EA also induced caspase-3-mediated apoptosis by increasing the Bax : Bcl-2 ratio and restored anoikis in both cell lines. The enhancement of apoptosis and/or inhibition of autophagy in these cells by EA assisted the chemotherapy efficacy. The results indicated that EA is a potential novel chemoprevention and treatment assistant agent for human ovarian carcinoma.

## 1. Introduction

Ovarian carcinoma is the most lethal gynecological cancer worldwide and the fifth most frequent cause of cancer death from all types of cancer in USA women, although it accounts for 3% to 4% of all cancer in women. Because of the nonspecific or lack of symptoms of early stage ovarian carcinoma, the majority of ovarian carcinoma patients are diagnosed with advanced stage disease [1, 2]. Patients with advanced stage ovarian carcinoma are treated with standard therapy including cytoreductive surgery and combination therapy. Approximately 75% of patients respond to the initial treatment; however, these patients will suffer from disease recurrence with fatal drug resistance [3]. Rapid relapse and the development of drug-resistant disease are the major challenges in ovarian carcinoma treatment. A novel strategy for the treatment of ovarian carcinoma is needed.

Ellagic acid (EA) is a polyphenol compound widely found in fruits such as berries, walnuts, pecans, pomegranate, cranberries, and longan. It is well known to have a free radical scavenging activity and has been approved in Japan as an “existing food additive” for antioxidative purposes [4]. Recent *in vitro* evidences revealed that 100 *μ*M EA represented little toxic effect on human normal cells [5, 6]. A 90-day subchronic toxicity study further demonstrated that orally feeding EA (9.4, 19.1, 39.1 g/kg b.w., resp.) could not induce mortality or treatment-related clinical signs throughout the experimental period on F344 rats [7], indicating the low toxicity of EA to mammalians. Furthermore, EA exhibits potent anticancer and anticarcinogenesis activities towards breast, colorectal, oral, prostate [5, 6, 8], pancreatic [9], bladder [10], neuroblastoma [11], melanoma [12], and lymphoma cells [13]. However, there is no evidence regarding the effect of EA on ovarian carcinoma. This study first provides cellular and molecular evidence for EA in terms of the inhibition of cell proliferation, anchorage independency (anoikis resistance), and inducing cell cycle arrest and apoptosis in two ovarian carcinoma cell lines. We also identified a synergistic combination effect of EA and doxorubicin by regulating autophagy and/or apoptosis. This study revealed the potential role of this polyphenol in ovarian carcinoma chemotherapy and chemoprevention.

## 2. Materials and Methods

### 2.1. Materials

Roswell Park Memorial Institute (RPMI) media 1640, minimum essential medium (MEM), fetal bovine serum (FBS), L-glutamine, trypsin, and antibiotics were purchased from Gibco Ltd. (Paisley, UK). Ellagic acid (EA), 3-(4,5-cimethylthiazol-2-yl)-2,5-diphenyl tetrazolium bromide (MTT), proteinase inhibitor cocktail, sodium orthovanadate, sodium fluoride, sodium pyrophosphate, Triton X-100, ammonia persulfate, acridine orange, *N,N,N*′*,N*′-tetramethylethylenediamine (TEMED), and Tween 20 were obtained from Sigma (St. Louis, MO). Bicinchoninic acid (BCA) protein assay reagent was purchased from Pierce (Rockford, IL). Acrylamide was obtained from Bio-Rad (Hercules, CA). Polyvinylidene fluoride (PVDF) membrane (Immobilon-P) was obtained from Millipore (Bedford, MA). Mouse monoclonal anticaspase 3, B cell lymphoma 2 (Bcl-2), cyclin A, cyclin D1, cyclin E, and cyclin B1 antibodies were obtained from Zymed (San Francisco, CA). Goat polyclonal anti-p53, Cip1/p21, poly[ADP-ribose] polymerase (PARP) and Bcl-2 associated protein X (Bax) antibodies, and goat anti-rabbit, anti-mouse, and rabbit anti-goat secondary antibodies conjugated with horseradish peroxidase (HRP) were purchased from R&D Systems (Minneapolis, MN). X-ray film was obtained from Fuji (Tokyo, Japan). Annexin V conjugated with FITC was obtained from Becton Dickinson (Franklin Lakes, NJ).

### 2.2. Cell Lines

Human ovarian carcinoma cell lines ES-2, PA-1, and lung fibroblast cell line MRC-5 were obtained from the Bioresource Collection and Research Center, Taiwan. ES-2 cells, which possess moderate resistance to doxorubicin, were established from a poorly differentiated carcinoma of the ovary taken from a black woman. ES-2 cells were cultured in 90% RPMI medium 1640 supplemented with 10% heat-inactivated FBS. PA-1 cells, established by culturing ascitic fluid cells from a Caucasian female with ovarian carcinoma, were cultured in 90% MEM medium supplemented with 2 mM L-glutamine, 1.5 g/L sodium bicarbonate, 0.1 mM nonessential amino acids, 1.0 mM sodium pyruvate, and 10% heat-inactivated FBS. MRC-5 were cultured in the same medium as PA-1. All media used here were supplemented with 25 U/mL penicillin and 25 *μ*g/mL streptomycin as the antibiotics. The cells were incubated at 37°C in a 95% air/5% CO_2_ and water-saturated atmosphere. All experiments were carried out on cell lines passaged 5–20 times.

### 2.3. Cell Proliferation Assay

Ovarian carcinoma cell lines were plated at 100,000 cells in six-well tissue culture dishes. After 18 h of culture, cells were treated with different concentrations of DMSO-dissolved EA (0, 10, 25, 50, 75, or 100 *μ*M) or chemotherapeutic drugs (doxorubicin, paraplatin, and paclitaxel) or a combination of both drugs. At the various time points, cells were collected by trypsinization and stained with trypan blue, and the cell number in suspension was counted in duplicate using a hemocytometer. The combination effect was calculated according to the equation described by Kern et al. [14]. The expected value for cell survival, *S*_exp⁡_, was defined as the product of the survival observed for EA alone and the survival observed for chemotherapeutic drug alone as follows:

(1) $$\begin{array}{c} S_{\exp}=S_{\text{EA}}\times S_{\text{chemo-drug}}. \end{array}$$

The actual survival observed for the combination of EA and chemotherapeutic drug was defined as *S*_obs_. A synergistic ratio, *R*, was calculated as

(2) $$\begin{array}{c} R=\frac{S_{\exp}}{S_{\text{obs}}}. \end{array}$$

Synergy was defined as any value of *R* greater than unity. Values of *R* of 1.0 or less indicated an absence of synergy.

### 2.4. Anoikis

Two methods were used to assess the anoikis of the ovarian carcinoma cells. First, we suspended ovarian carcinoma cells (5 × 10^5^ cells) and seeded them in a low cell attachment six-well tissue culture dish (Costar no. 3471) and then treated the cells with different concentrations of EA. Two days after treatment, the formed cell clusters were assessed under a microscope. The clustered cells were scattered and the surviving cells were stained with trypan blue and counted under a microscope. The apoptotic cells were assessed by staining cells with annexin V and analyzing by flow cytometry, as described in Section 2.6. Second, ovarian carcinoma cells were plated in soft agar for colony formation. Briefly, a feeder layer of medium supplemented with 20% serum and 0.6% agarose was plated in a 60 mm tissue culture dish and overlaid with a layer of 3000 cells in medium with 20% serum and 0.3% agarose containing one of several concentrations of EA. The dishes were incubated at 37°C for 14 days. Colonies were stained with 2 mg/mL MTT followed by culturing at 37°C for an additional 4 hours, and then the colonies were counted using a stereomicroscope.

### 2.5. Cell Cycle Analysis

The cell cycle distribution of EA-treated cells was measured by the DNA content in each cell using flow cytometry as described by Chung et al. [15]. Briefly, treated cells were harvested by trypsinization, fixed in 70% ethanol at −20°C for at least 30 min, and then stained with propidium iodide solution (20 *μ*g/mL propidium iodide and 10 *μ*g/mL RNase A) at 37°C in the dark for 30 min. The stained cells were examined by flow cytometry (FASCalibur, Becton Dickinson) using FL-2A to score the DNA content of the cells. The percentages of cells in the G1, S, and G2/M cell cycle phases were determined using Modfit software (Verity Software House, Inc., Topsham, ME).

### 2.6. Apoptosis Analysis

Apoptosis measurement was carried out using annexin V to label cell-surface phosphatidylserine of apoptotic cells, as described in Chung et al. [15]. Briefly, treated cells were trypsinized and washed twice with phosphate-buffered saline and then suspended in binding buffer (10 mM HEPES, pH 7.4, 140 mM NaCl, and 2.5 mM CaCl_2_). Cells were stained with a final concentration of 2 *μ*g/mL of annexin V conjugated with fluorescein isothiocyanate (FITC) at room temperature in the dark for 30 min. The stained cells were measured by a flow cytometry using FL-1H as the parameter to monitor the apoptosis cells. Untreated cells served as the negative control.

### 2.7. Autophagy

Autophagolysosome formation of EA-treated ovarian carcinoma cells was assessed using acridine orange staining of the cells and subsequent analysis under a confocal fluorescence microscope and by flow cytometry, essentially as described by Böck et al. [16]. Briefly, EA- or doxorubicin-treated ovarian carcinoma cells were stained in complete medium containing 1 *μ*g/mL acridine orange and the autophagolysosomes were examined under a confocal fluorescence microscope (Zeiss Observer Z1, Carl Zeiss International, Germany) using a 488 nm wavelength laser as the excitation light. Cells treated in the same manner were also trypsinized and stained with 1 *μ*g/mL acridine orange for 30 min and then analyzed by flow cytometry using FL-3H as the parameter to monitor the autophagolysosome change. 

### 2.8. Immunoblotting

Cells were washed with ice-cold phosphate-buffered saline and lysed in homogenization buffer (10 mM Tris-HCl at pH 7.4, 2 mM EDTA, 1 mM EGTA, 50 mM NaCl, 1% Triton X-100, 50 mM NaF, 20 mM sodium pyrophosphate, 1 mM sodium orthovanadate, and 1 : 100 proteinase inhibitor cocktail) on ice for 30 min. After centrifugation for 30 min at 13,000 rpm at 4°C to remove insoluble materials, the protein concentration of the lysate was determined using a BCA protein assay kit, and the lysate was then separated using SDS-PAGE. The resolved bands were electrotransferred to PVDF membranes using a semidry blot apparatus (Bio-Rad). Immunoblotting was performed by incubating PVDF membranes with 5% nonfat milk in Tris-buffered saline supplemented with Tween 20 (TBST, 10 mM Tris, pH 7.4, 150 mM NaCl, 0.2% Tween 20) for 1 h at room temperature to block the residue free protein binding sites on PVDF. The membrane was incubated with different primary antibodies in 3% non-fat milk in TBST at 4°C for 18 h. After repeated washing with TBST, the membrane was incubated with secondary antibodies conjugated with HRP. Immunoblots were developed using enhanced chemiluminescence and the luminescence was visualized on X-ray film or using a chemoluminescence detection system (Bio-Rad).

### 2.9. Statistical Analysis

All data were averaged from three independent experiments and are expressed as the means ± standard deviation (SD) unless stated otherwise. Differences between groups were calculated using Student's unpaired *t*-test. Dose-dependent effect was calculated using simple linear regression. *P* < 0.05 was considered statistically significant. All statistical analyses were performed using SPSS version 12.0 (SPSS, Inc., Chicago, IL).

## 3. Results

### 3.1. Inhibition of Ovarian Carcinoma Cell Proliferation

**Figure 1:**
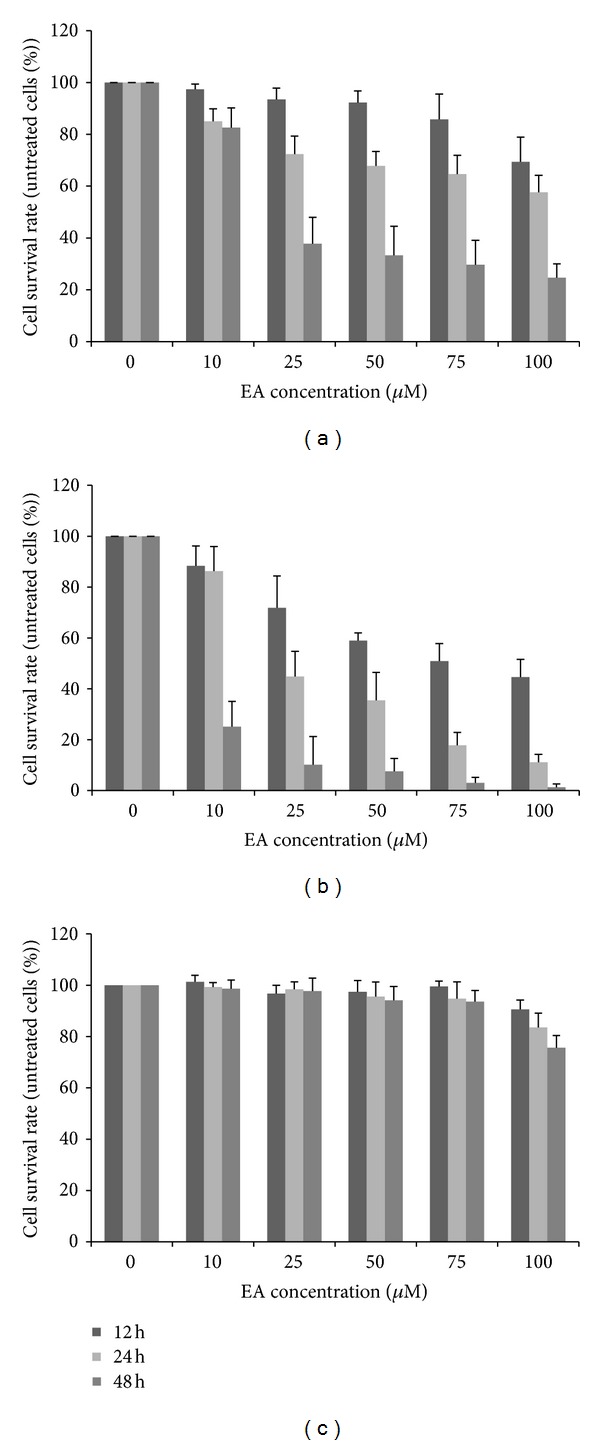
The dose and time response of ovarian carcinoma cell lines to ellagic acid (EA). A total of 100,000 ES-2 cells (a), PA-1 cells (b), and MRC-5 cells (c) in 6-well plates were treated with increasing concentrations of EA as indicated and then incubated at 37°C for 12, 24, and 48 h. Viable cells were trypsinized, stained with trypan blue, and counted under a microscope. Cell viability was expressed as a percentage of untreated cells. Data are the average of three independent experiments and expressed as means ± SD.

As shown in Figure 1, compared with untreated cells, survival decreased in a dose-dependent and time-dependent manner (*P* < 0.05) in both ovarian carcinoma cells. ES-2 cell growth was significantly suppressed under greater than 25 *μ*M EA treatment after 48 hours, with more than 60% inhibition, although there was unremarkable change at 12 and 24 hours (Figure 1(a)). PA-1 was more sensitive to EA. The cell proliferation of PA-1 was suppressed under 25 *μ*M EA treatment after 12 hours with 30% inhibition. More than 70% inhibition at 10 *μ*M and 90% inhibition at 25 *μ*M at 48 hours were observed (Figure 1(b)). All the tested concentration of EA except 100 *μ*M could not inhibit the growth of human lung normal fibroblast cell line MRC-5 (Figure 1(c)).

### 3.2. G1 Phase Arrest

**Figure 2:**
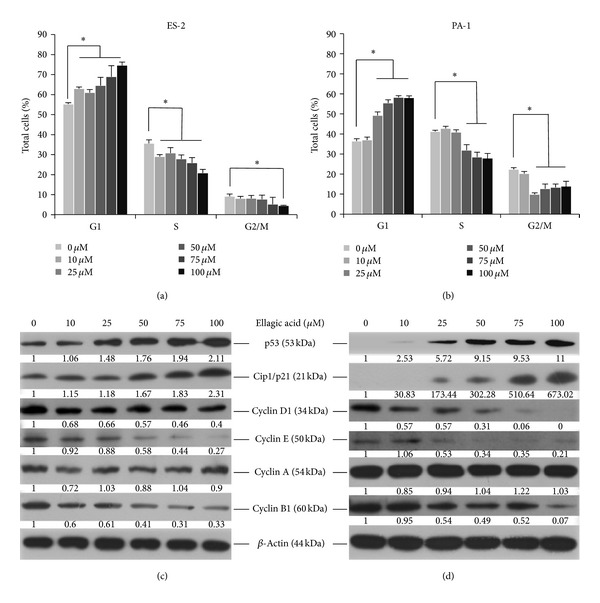
Cell-cycle arrest in ovarian carcinoma cells by EA. EA-treated cells were incubated at 37°C for 12 h and then fixed in 70% alcohol and stained with propidium iodide then analyzed by flow cytometry, as described in Section 2. The same EA-treated cells were lysed and the cell proteins were separated by SDS-PAGE then immunoblotted to show proteins as indicated with the beta-actin level as the loading control. Protein levels were quantified using Image Lab software (Bio-Rad) according the density of each band on the immunoblotting image, normalized to the reference band (*β*-actin) and presented as the fold of the untreated control. The cell-cycle distribution and the changes in cell-cycle-associated proteins of EA-treated ES-2 are shown in (a) and (c), respectively, and EA-treated PA-1 are shown in (b) and (d). The data reported are the percentages of total cells from the averages of three independent experiments and are expressed as means ± SD. *P < 0.05.

As shown in Figure 2, the distribution of the G1 phase of the cell cycle gradually increased and the S and G2/M phases concomitantly decreased at EA concentrations greater than 10 *μ*M in ES-2 cells (Figure 2(a)) and 25 *μ*M in PA-1 cells (Figure 2(b)) when compared with untreated controls. To examine the protein mechanisms by which EA induced G1 phase block in ovarian carcinoma cells, the levels of cell-cycle-controlling proteins cyclin D1, E, A, and B1 and cyclin dependent protein kinase inhibitor Cip1/p21 and tumor suppressor p53 were determined by immunoblotting. As shown in Figure 2(c), untreated ES-2 expressed p53, and the level of p53 was elevated under treatment with more than 25 *μ*M EA to 1.4- to 1.6-fold of the control level. Cip1/p21 levels were increased in ES-2 cells under treatment with more than 10 *μ*M EA, whereas cyclin B1 levels were decreased in a dose-dependent manner. Cyclin D1 levels were decreased in ES-2 cells under treatment with more than 10 *μ*M EA whereas cyclin E decreased with more than 50 *μ*M EA. The change in the cyclin A level of the EA-treated ES-2 cells was unremarkable. The profile of the changes in cell cycle proteins in EA-treated PA-1 was more dramatic than that in ES-2 cells. As shown in Figure 2(d), untreated PA-1 cells expressed trace levels of p53, and the level elevated dramatically under treatment with more than 25 *μ*M EA to 2.53- to 11-fold of the control level. Cip1/p21 levels were significantly expressed under treatment with more than 25 *μ*M EA to 30.83- to 673.02-fold of control level, whereas cyclin D1, E, and B were gradually decreased. Cyclin A showed no remarkable change. 

### 3.3. Apoptotic Death

**Figure 3:**
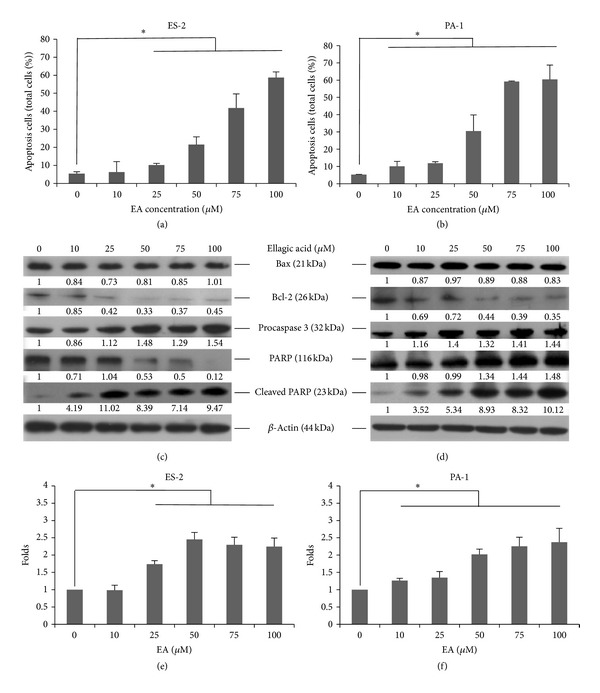
EA-induced apoptosis in ovarian carcinoma cells. EA-treated cells were incubated at 37°C for 12 h and stained with annexin V conjugated with FITC and then analyzed by flow cytometry. Cell protein lysates from EA-treated cells were separated by SDS-PAGE, transferred to PVDF membranes, and immunoblotted to show proteins as indicated. Protein levels were quantified and normalized using the density of the untreated control, and the Bax : Bcl-2 ratio was calculated. The apoptotic cells, the changes in apoptosis-associated proteins, and the Bax : Bcl-2 ratio of EA-treated ES-2 are shown in (a), (c), and (e), respectively, and EA-treated PA-1 are shown in (b), (d), and (f). The data reported are the averages of three independent experiments and are expressed as means ± SD. *P < 0.05.

Phosphatidylserine translocation was assessed to determine apoptosis in EA-treated ovarian carcinoma cells by staining with FITC-conjugated annexin V. Annexin V-positive ES-2 cells increased under treatment with more than 25 *μ*M EA (Figure 3(a); *P* < 0.05), whereas PA-1 cells increased under treatment with 10 *μ*M EA (Figure 3(b)), compared with untreated cells. Procaspase 3 increased and Bcl-2 decreased under treatment with more than 25 *μ*M EA in ES-2 cells and 10 *μ*M EA in PA-1 cells (Figures 3(c) and 3(d)). The caspase 3 substrate PARP was cleaved in both EA-treated ES-2 and PA-1 cells. Bax levels were not changed remarkably. The Bax : Bcl-2 ratio was increased under treatment with more than 25 *μ*M EA in ES-2 cells and 10 *μ*M EA in PA-1 cells (Figures 3(e) and 3(f)).

### 3.4. Anoikis Induction

**Figure 4:**
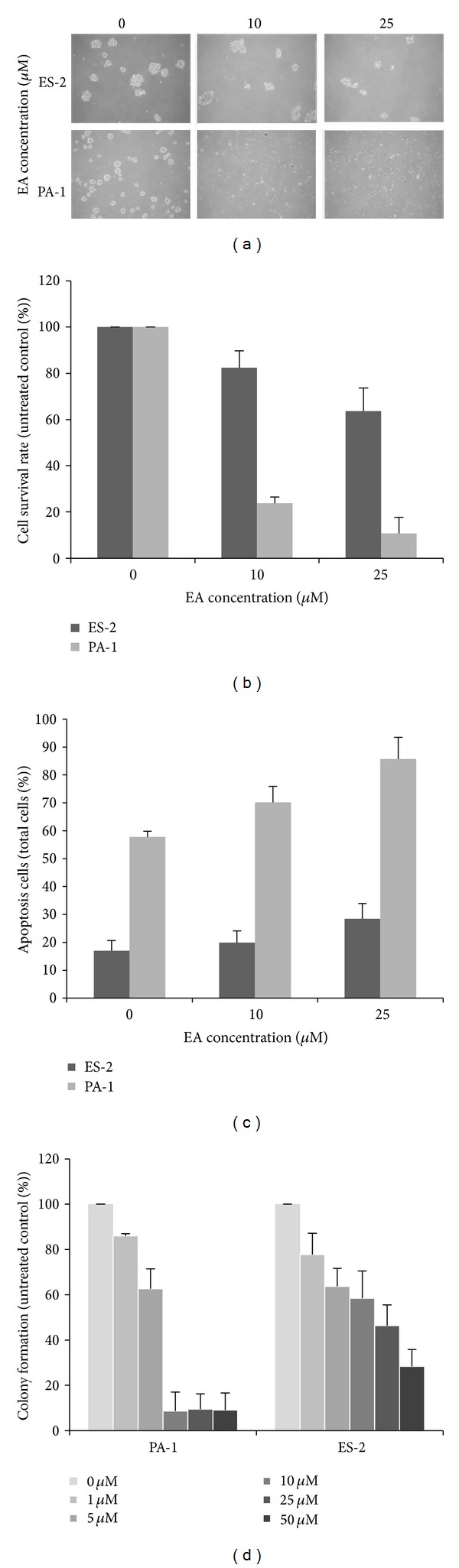
Anchorage-independent growth and anoikis in EA-treated ovarian carcinoma cells. Cells were cultured in methylcellulose-coated 6-well plates and treated with different concentrations of EA as indicated. The formed cell clusters were photographed under a microscope (a). The suspended cell clusters were scattered and stained with trypan blue. The surviving cells were counted using a haemocytometer (b). The same scattered cells were stained with annexin V-FITC and then subjected to flow cytometry for apoptosis analysis (c). Cells were suspended in soft agar and incubated with different concentrations of EA as indicated for 14 days for anchorage-independent growth in soft agar (d). The data reported are the averages of three independent experiments and are expressed as means ± SD.

Resistance to anoikis, which is similar to anchorage-independent growth, is the malignant phenotype of cancer cells closely correlated with the *in vivo* tumorigenesis ability in nude mice [17]. The first assay was carried out by seeding cells into low attachment tissue culture dishes, in which the cells were difficult to attach to the bottom of the plates, and living cells therefore form spheroid cell clusters. In contrast to living cells, the dead cells dispersed and became apoptotic. As shown in Figure 4(a), ES-2 and PA-1 cells were significantly reduced cell clusters under treatment with EA greater than 25 *μ*M and 10 *μ*M, respectively, whereas untreated ES-2 and PA-1 cells underwent spheroid formation. The survival rate of ES-2 cells in the suspension culture was decreased to 82% and 65% of the control level, respectively, under 10 *μ*M and 25 *μ*M EA treatment (Figure 4(b)). PA-1 cells were more sensitive to EA treatment in suspension culture, with 23% and 12% of the cells surviving under 10 *μ*M and 25 *μ*M EA treatment. We also analyzed the apoptotic cells in a suspension culture of these two cells and found that ES-2 cells consisted of lesser cells in apoptosis, at 18%, 21%, and 27% under 0, 10, and 25 *μ*M EA treatment. In contrast, detached PA-1 cells showed more apoptotic cells, at 57%, 71%, and 86% under 0, 10, and 25 *μ*M EA treatment (Figure 4(c)). The other anchorage-independent growth assay was carried out by suspending cells in soft agar. As shown in Figure 4(d), the formation of colonies in soft agar was dramatically decreased under treatment with more than 10 *μ*M EA in PA-1 cells whereas the colony formation of ES-2 cells gradually decreased with increasing EA concentration.

### 3.5. Autophagy and Chemoassistant Effect

**Figure 5:**
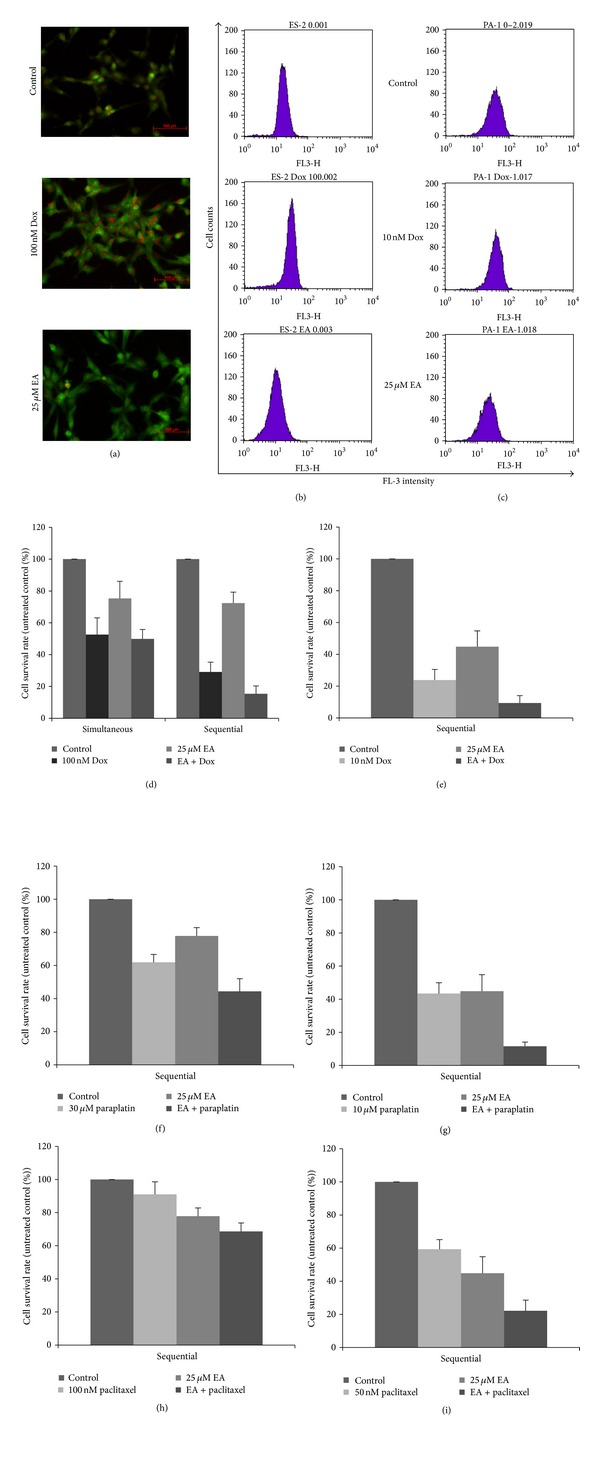
Autophage-inhibiting effect and chemoassistance of EA. Cells were treated with or without EA or doxorubicin for 24 hours and stained with acridine orange. The macrophagosomes of ES-2 cells were monitored under a confocal microscope using a 488 nm wavelength light to stimulate fluorescence (a). The treated cells, stained with acridine orange, were subjected to flow cytometry, and the FL-3 intensity was scored (b). The acridine orange-stained PA-1 cells were analyzed by flow cytometry (c). ES-2 cells were seeded in 6-well plates and treated with EA, doxorubicin, or a simultaneous combination of these two drugs for 24 hours (Simultaneous), or pretreated with doxorubicin for 24 hours and then treated with EA for another 24 hours (Sequential). Cells were trypsinized, stained with trypan blue, and counted under a hemocytometer (d). PA-1 cells were seeded in 6-well plates and pretreated with doxorubicin for 24 hours and then treated with EA for another 24 hours (e). ES-2 cells pretreated with paraplatin and paclitaxel following by EA were shown in (f) and (h), respectively. PA-1 cells pretreated with paraplatin and paclitaxel following by EA were shown in (g) and (i), respectively. The data reported are the averages of three independent experiments and are expressed as means ± SD.

Autophagy, a homeostatic, catabolic degradation process, prevents the accumulation of damaged proteins or organisms. This process could be triggered in cancer cells, recycling substances and producing metabolic energy, which may facilitate rapid tumor growth and chemoresistance [18]. We attempted to analyze whether EA could influence this process and assess the possible role in ovarian carcinoma cell chemoresistance to doxorubicin. As shown in Figure 5(a), ES-2 cells, with doxorubicin resistance, possessed some autophagolysosomes in their cytoplasm. The intensity and numbers of autophagolysosomes were increased in doxorubicin-treated ES-2 cells and decreased in EA-treated cells. Flow cytometry analysis also showed that the FL-3 intensity of acridine orange-stained ES-2 cells was in the range from 50 to 500 arbitrary units, with the mean value at 180. The mean value was shifted to 300 when ES-2 cells were treated with 100 nM doxorubicin for 24 hours. The mean value was shifted to 100 when ES-2 cells were treated with 25 *μ*M EA (Figure 5(b)). The autophage induction of PA-1 cells by doxorubicin was not remarkable whereas EA-treated PA-1 cells showed suppressed autophagy (Figure 5(c)). Simultaneously treating ES-2 cells with 100 nM doxorubicin and 25 *μ*M EA for 24 hours resulted in an unremarkable inhibition effect compared with 100 nM doxorubicin treatment. Calculation of the combination index showed an antagonistic effect (*R* = 0.796). When ES-2 cells were pretreated with 100 nM doxorubicin for 24 hours and then treated with 25 *μ*M EA for another 24 hours, a synergistic effect was found (*R* = 1.419; Figure 5(d)). A synergistic effect of pretreatment with doxorubicin followed by EA treatment was also found in PA-1 cells (Figure 5(e)). Similar effects of pretreatment with paraplatin or paclitaxel followed by EA treatment were also found in both ovarian carcinoma cell lines (Figures 5(f), 5(g), 5(h), and 5(i)).

## 4. Discussion

In this study, we investigated the effects of EA treatment on ES-2 and PA-1 ovarian carcinoma cells. EA inhibited proliferation of these two cell lines in a dose- and time-dependent manner. EA could not influence the growth of normal cell line MRC-5 at less than 75 *μ*M EA treatment, indicating that EA represents the selective inhibition effect of cell growth on carcinoma cells. The inhibition effect of EA on these two cells arose from cell cycle arrest at the G1 phase, which was mechanistically associated with the elevation of p53 and Cip1/p21 levels and the suppression of cyclin D1 and E. EA also induced these two cells to apoptotic death by upregulating the Bax : Bcl-2 ratio and caspase 3 activation. Anoikis of these two cancer cells was restored by EA treatment. EA treatment suppressed autophagy and the other effects described earlier were the possible mechanisms to potentiate the sensitivity of ovarian carcinoma cells to chemotherapeutic drug treatment. To the best of our knowledge, this is the first direct evidence that EA can inhibit the proliferation, malignant potential, and chemoresistance of ovarian carcinoma cells. 

EA can inhibit cell proliferation and cell cycle progression or induce apoptotic death of breast, colorectal, oral, prostate [5, 6, 8], pancreatic [9], bladder [10], neuroblastoma [11], melanoma [12], and lymphoma cells [13] by regulating intracellular molecules that control cell growth and survival. Mertens-Talcott and colleagues revealed that EA potentiated the effect of quercetin on elevating the Cip1/p21 and p53 levels in Molt-4 leukemic lymphoma cells [19]. Li and colleagues further reported that EA induced G1 phase arrest and apoptosis by directly elevating the Cip1/p21 and p53 expression levels in a bladder cancer T24 cell line [10]. In this study, we found that EA also promoted the expression of these two tumor suppressors in both ES-2 and PA-1 cells. Theoretically, damaged DNA of the cells could induce *de novo* synthesis of tumor suppressor p53. The expressed p53 activates Cip1/p21 expression to perform repair of DNA damage or Bax expression to induce apoptotic death of cells [10]. As our results presented herein, the p53 level was gradually elevated by increasing the concentration of EA treatment in both cell lines, indicating that EA may induce DNA damage in these two ovarian carcinoma cells. The elevation of the Cip1/p21 level in these two cell lines was correlated with the change in the p53 level, and the expression level of the apoptosis protein Bax was slightly suppressed in EA-treated ovarian carcinoma cells. This may lead the ovarian carcinoma cells to arrest at the G1 phase of the cell cycle and facilitate the repair of DNA damage [20]. Other factors that influence EA-treated ovarian carcinoma cells to be arrested at the G1 phase may arise from the downregulation of cyclins. Both EA-treated ovarian carcinoma cells possessed decreasing levels of cyclin D1, E, and B1. Cyclin D1 and E are two major regulators of CDK4/6 activity, which could phosphorylate/inactivate retinoblastoma protein and activate E2F transcriptional factor to trigger the expression of DNA synthesis enzymes [21]. In this study, suppressed cyclin D1 and E levels by EA treatment were observed in both ovarian carcinoma cells, indicating their possible role in EA-induced G1 phase arrest. Cyclin B1, which associates with cdc2 and leads cells to enter mitosis, was decreased in both EA-treated ovarian carcinoma cells, similar to the result observed in Caco-2 colorectal cancer cells [6]. Theoretically, decreasing the cyclin B1 level leads cells to arrest at the G2/M phase of the cell cycle [22]. However, our results did not adhere to this theory. One recent report from Vuletic and colleagues revealed that 13-cis-retinoic acid-treated HL-60 leukemia cells exhibited G0/G1 arrest and decreased cyclin B1 [23]. One possible reason may be the high level of cyclin B1 in G2/M phase cells, while G1 or S phase cells possess low levels of cyclin B1 [24]. As the population of EA-treated ovarian carcinoma cells was most in the G1 phase, the cyclin B1 level may be diluted and gradually decreased by increasing the G1 phase cells. From these results, we conclude that the G1 phase arrest of EA-treated ovarian carcinoma cells arises mainly from upregulation of p53 and Cip1/p21 and the suppression of cyclin D1 and E.

EA-treated ovarian carcinoma cells also showed early apoptosis, exhibiting increasing annexin V-positive cells. The main reason may be the suppression of the antiapoptosis protein Bcl-2 level by EA treatment. Bcl-2 expression was found to be associated with the resistance of ovarian carcinoma to chemotherapy drugs [25]. In our recent report, the ratio of Bax : Bcl-2 proteins was found to be the determining factor in transmitting the apoptosis signal, which finally triggered caspase-3 activation [15]. The present study showed that the Bcl-2 level gradually decreased as the concentration of EA treatment increased in both ovarian carcinoma cells, and the Bax : Bcl-2 ratio also increased, which was associated with caspase 3 activation and the cleavage of caspase 3 substrate PARP and apoptotic induction. We could conclude therefore that the inhibition of ovarian carcinoma cell proliferation arose mainly from the combination effect of p53/p21-mediated G1 phase arrest of the cell cycle and Bax/Bcl-2 ratio/caspase-3-induced apoptotic death.

Anchorage-independent growth is the ability by which cells can survive under detached circumstances. Differentiated epidermal cells are restricted in terms of this ability and must attach to the basal membrane of epidermal tissues to survive. Once the epidermal cells detach from the basal membrane, they will be eliminated by apoptosis; this is specifically named anoikis [26]. The acquisition of anchorage independence is an important hallmark of malignant transformation and is thought to be one of the critical factors in the metastasis and dissemination of ovarian cancer [27]. Our previous study revealed that suppression of anchorage-independent growth was correlated with* in vivo* tumorigenesis of cancer cells [17]. Overcoming the anchorage independence of cancer cells is a promising strategy for cancer therapy [28]. Herein, we found that ovarian carcinoma cells PA-1 and ES-2 treated with EA lose their anchorage independence, which was assessed by two different anchorage independence assays (see Figure 4). However, a differing response in terms of anoikis induction between these two cells to EA treatment was also found. The numbers of PA-1 clusters and surviving cells were dramatically decreased under 10 *μ*M EA treatment and the cells showed anoikis. However, the same treatment could only reduce the total cluster number and only partially restrict the anchorage independence of ES-2 cells. ES-2 cells still survived and formed clusters under 25 *μ*M EA treatment, and anoikis of ES-2 cells was lesser apparent than that of PA-1 cells in the suspension culture. A similar result was also found regarding clonogenic growth in the soft agar assay. The colonies of PA-1 cells were dramatically decreased under 10 *μ*M EA treatment, while only the total number of colonies of ES-2 cells was reduced under the same condition. The different sensitivity of these two cells in terms of anoikis induction to EA treatment may arise from some other cellular and molecular mechanisms, and this is an intriguing issue for further investigation.

Autophagy is a homeostatic, catabolic process of lysosomal degradation by which cells recycle damaged proteins and organelles and provide energy and/or substance resources for cell reconstruction and facilitating survival during stress or starvation [16]. Tumor cells are capable of triggering autophagy to respond to stress and facilitate tumor progression and confer resistance to anticancer treatment. Targeting cytoprotective autophagy in cancers has the potential to enhance the treatment efficacy [18]. Accordingly, we proposed that the autophagy process may be part of the mechanism by which ES-2 cells resist chemotherapeutic drugs such as doxorubicin, since pegylated liposomal doxorubicin is an established treatment for relapsed ovarian cancer [29]. Since acridine orange is regarded as a lysosomotropic dye that could accumulate in acidic vesicles in a pH-dependent manner, the dye is used to detect autophagolysosome and autophagy recently. At neutral pHs, acridine orange is a weak base without charge that moves freely across biological membrane and shows green fluorescence with excitation light (488 nm). Within acidic vesicles, its protonated form is trapped within the organelle, where its aggregates emit bright red fluorescence [30]. Our results first demonstrated that autophagolysosomes, stained with acridine orange, were elevated in 24-hour 100 nM doxorubicin-treated ES-2 cells, of which the survival rate was 52.6%. The survival rate of ES-2 was 34.7% after 48-hour100 nM doxorubicin treatment. Compared with the survival rate was 48 hour-10 nM doxorubicin-treated PA-1, of which was 23.8%, ES-2 cells represented more resistant to doxorubicin treatment. The autophagolysosomes decreased in only EA-treated ES-2 cells, which indicated the autophagy-inhibiting effect of EA in ES-2 cells. However, the combination effect of simultaneously treating ES-2 cells with doxorubicin and EA was no addition effect. Reactive oxygen species are involved in one of the important cytotoxic mechanisms of doxorubicin towards cancer cells [31]. EA possesses a potent antioxidation ability and may counteract doxorubicin-induced reactive oxygen species. We treated ES-2 cells with doxorubicin and EA sequentially and found a synergistic effect in ES-2 cells. A similar effect was also observed following sequential treatment of PA-1 cells with doxorubicin and EA. Since paraplatin and paclitaxel are the first line chemotherapeutic drugs to ovarian carcinoma and the relapse ovarian carcinoma are always resistant to these two agents. We also found the synergistic effects of pretreatment of paraplatin or paclitaxel followed by EA treatment in both ovarian carcinoma cells. These results indicate that pretreatment of chemotherapeutic drugs followed by EA treatment could overcome the chemoresistance of ovarian carcinoma. We proposed that the inhibition of autophagy or combination with apoptosis may play a possible role in amelioration of drug resistance by EA to ovarian carcinoma. The actual subcellular and molecular mechanisms by which EA inhibits autophagy assisting in overcoming drug resistance of ovarian carcinoma is an intriguing issue for further investigation.

In conclusion, this study demonstrated that EA treatment inhibits cell proliferation and malignant potential in two ovarian carcinoma cells, ES-2 and PA-1. EA inhibited ovarian carcinoma cells mainly through G1 phase arrest of the cell cycle, through elevating the levels of p53 and Cip1/p21 and suppressing the levels of cyclin D1 and E. EA also induced apoptosis by inhibiting the Bcl-2 level and altering the Bax : Bcl-2 ratio and activating caspase 3. EA potently elevated anoikis in PA-1 cells; however, ES-2 cells appeared to be less sensitive to EA-induced anoikis. The differing sensitivity to anoikis induction may be the main reason for which PA-1 cells were more sensitive to EA treatment than ES-2 cells. EA also inhibited autophagy of ES-2 cells and may be associated with an assisting effect in the chemotherapeutic drug efficacy. We found that EA treatment exhibited both antimalignance and antichemoresistance properties in ovarian carcinoma cell lines, suggesting its potential as a novel chemopreventive and chemoassistant agent for ovarian cancer.

## References

[1] Deraco M, Baratti D, Laterza B (2011). Advanced cytoreduction as surgical standard of care and hyperthermic intraperitoneal chemotherapy as promising treatment in epithelial ovarian cancer. *European Journal of Surgical Oncology*.

[2] Gómez-Raposo C, Mendiola M, Barriuso J, Hardisson D, Redondo A (2010). Molecular characterization of ovarian cancer by gene-expression profiling. *Gynecologic Oncology*.

[3] Cohen M, Dromard M, Petignat P (2010). Heat shock proteins in ovarian cancer: a potential target for therapy. *Gynecologic Oncology*.

[4] HHLW (Ministry of Health, Labor and Welfare of Japan)

[5] Losso JN, Bansode RR, Trappey A, Bawadi HA, Truax R (2004). In vitro anti-proliferative activities of ellagic acid. *Journal of Nutritional Biochemistry*.

[6] Larrosa M, Tomás-Barberán FA, Espín JC (2006). The dietary hydrolysable tannin punicalagin releases ellagic acid that induces apoptosis in human colon adenocarcinoma Caco-2 cells by using the mitochondrial pathway. *Journal of Nutritional Biochemistry*.

[7] Tasaki M, Umemura T, Maeda M (2008). Safety assessment of ellagic acid, a food additive, in a subchronic toxicity study using F344 rats. *Food and Chemical Toxicology*.

[8] Malik A, Afaq S, Shahid M, Akhtar K, Assiri A (2011). Influence of ellagic acid on prostate cancer cell proliferation: a caspase-dependent pathway. *Asian Pacific Journal of Tropical Medicine*.

[9] Edderkaoui M, Odinokova I, Ohno I (2008). Ellagic acid induces apoptosis through inhibition of nuclear factor *κ*B in pancreatic cancer cells. *World Journal of Gastroenterology*.

[10] Li T-M, Chen G-W, Su C-C (2005). Ellagic acid induced p53/p21 expression, G1 arrest and apoptosis in human bladder cancer T24 cells. *Anticancer Research*.

[11] Fjaeraa C, Nånberg E (2009). Effect of ellagic acid on proliferation, cell adhesion and apoptosis in SH-SY5Y human neuroblastoma cells. *Biomedicine and Pharmacotherapy*.

[12] Kim S, Liu Y, Gaber MW, Bumgardner JD, Haggard WO, Yang Y (2009). Development of chitosan-ellagic acid films as a local drug delivery system to induce apoptotic death of human melanoma cells. *Journal of Biomedical Materials Research*.

[13] Mishra S, Vinayak M (2011). Anti-carcinogenic action of ellagic acid mediated via modulation of oxidative stress regulated genes in Dalton lymphoma bearing mice. *Leukemia and Lymphoma*.

[14] Kern DH, Morgan CR, Hildebrand-Zanki SU (1988). In vitro pharmacodynamics of 1-*β*-D-arabinofuranosylcytosine: synergy of antitumor activity with cis-diamminedichloroplatinum(II). *Cancer Research*.

[15] Chung Y-C, Lin C-C, Chou C-C, Hsu C-P (2010). The effect of Longan seed polyphenols on colorectal carcinoma cells. *European Journal of Clinical Investigation*.

[16] Böck BC, Tagscherer KE, Fassl A (2010). The PEA-15 protein regulates autophagy via activation of JNK. *Te Journal of Biological Chemistry*.

[17] Chung Y-C, Chang K-J, Yang C-C (2002). Association of proline-directed protein kinase FA with tumorigenesis, invasion, and poor prognosis of human colon carcinoma. *Cancer*.

[18] Yang ZJ, Chee CE, Huang S, Sinicrope FA (2011). The role of autophagy in cancer: therapeutic implications. *Molecular Cancer Therapeutics*.

[19] Mertens-Talcott SU, Bomser JA, Romero C, Talcott ST, Percival SS (2005). Ellagic acid potentiates the effect of quercetin on p21 waf1/cip1, p53, and MAP-kinases without affecting intracellular generation of reactive oxygen species in vitro. *Journal of Nutrition*.

[20] Weiss RH (2003). p21Waf1/Cip1 as a therapeutic target in breast and other cancers. *Cancer Cell*.

[21] Zhang K, Kumar R (1994). Interferon-*α* inhibits cyclin E- and cyclin D1-dependent CDK-2 kinase activity associated with RB protein and E2F in Daudi cells. *Biochemical and Biophysical Research Communications*.

[22] Taylor WR, Stark GR (2001). Regulation of the G2/M transition by p53. *Oncogene*.

[23] Vuletic A, Konjevic G, Milanovic D, Ruzdijic S, Jurisic V (2010). Antiproliferative effect of 13-cis-retinoic acid is associated with granulocyte differentiation and decrease in cyclin B1 and BCL-2 protein levels in G0/G1 arrested HL-60 cells. *Pathology and Oncology Research*.

[24] Miyazaki T, Arai S (2007). Two distinct controls of mitotic Cdk1/cyclin B1 activity requisite for cell growth prior to cell division. *Cell Cycle*.

[25] Mano Y, Kikuchi Y, Yamamoto K (1999). Bcl-2 as a predictor of chemosensitivity and prognosis in primary epithelial ovarian cancer. *European Journal of Cancer*.

[26] Gilmore AP (2005). Anoikis. *Cell Death and Differentiation*.

[27] Nishikawa T, Tsuno NH, Okaji Y (2010). Inhibition of autophagy potentiates sulforaphane-induced apoptosis in human colon cancer cells. *Annals of Surgical Oncology*.

[28] Hannigan G, Troussard AA, Dedhar S (2005). Integrin-linked kinase: a cancer therapeutic target unique among its ILK. *Nature Reviews Cancer*.

[29] Parma G, Mancari R, Del Conte G (2012). An open-label phase 2 study of twice-weekly bortezomib and intermittent pegylated liposomal doxorubicin in patients with ovarian cancer failing platinum-containing regimens. *International Journal of Gynecological Cancer*.

[30] Paglin S, Hollister T, Delohery T (2001). A novel response of cancer cells to radiation involves autophagy and formation of acidic vesicles. *Cancer Research*.

[31] Fang J, Nakamura H, Iyer AK (2007). Tumor-targeted induction of oxystress for cancer therapy. *Journal of Drug Targeting*.

